# Chronic Inflammation of the Os and Cervix Uteri

**Published:** 1870-01

**Authors:** Noble Holton

**Affiliations:** Buda, Ill.


### CHICAGO MEDICAL EXAMINER.

N. S. DAVIS, M.D., Editor.

VOL. XI. JANUARY, 1870. NO. 1.

### Original Contributions.

### ARTICLE I.

### CHRONIC INFLAMMATION OF THE OS AND CERVIX UTERI.

By NOBLE HOLTON, M.D., Buda, Ill.

It is not uncommon for us to be called to see ladies, who are out of health, and not confined to their beds constantly, and yet suffer to such an extent as to make them say, oftentimes, that they wish they could be relieved, even by death. They are hardly able to give expression to their feelings, or to give any symptoms, or aggregation of symptoms, that would account rationally for their feelings. By careful investigation, however, we can draw out a rational and connected history, pointing to the uterus, as the organ primarily at fault. The digestive organs are as soon noticed to be in an abnormal condition, perhaps, as any other. This is not at all surprising; for the physiological condition known as pregnancy produces remarkable effects on the digestive organs, and is not a disease at all, but does it through sympathetic nervous action. Anorexia, flatulency, constipation, gastralgia, and sometimes nausea, with loathing of food, are some of the symptoms in connection with the digestive organs.

These symptoms vary in degree, from the slightest functional disturbance to the greatest intensity; and just in proportion to the violence of these symptoms will be the chlorotic or anæmic condition of the patient. Constipation is an early and very damaging symptom, for it is not only a source of irritation, by its proximity to the uterus, but, by the straining necessary to evacuate the bowels, when loaded with hardened feces, the effort forces the os uteri against the distended rectum, thus greatly increasing the disease of the uterus.

Some ladies describe the pain of such action of the bowels to be about as bad as labor at full time. Under this condition of the bowels, the womb is forced down and its ligaments stretched out, each time the bowels are emptied, till they become relaxed to such an extent as to allow the os to rest against the rectum constantly.

The resulting irritation soon sets up or increases the inflammation, thus increasing its weight, and the weight increasing the prolapsus, and the prolapsus increasing the constipation, by mechanical obstruction.

Constipation is quite a constant symptom in chronic inflammation of the os and cervix uteri, and generally depends upon want of tone in the muscular structure of the bowels; but occasionally the inflammation extends to the mucous membrane of the bowels, to a sufficient extent to produce a mucous diarrhœa, from irritation, or, otherwise, to alternate constipation and diarrhœa, or to cause the formation of tube casts of the rectum, of muco-fibrinous character. All these conditions are accompanied with tumultuous movement of gas in the bowels and stomach, or with annoying distension or borborigmus, at times. In some cases, particular kinds of food appear to aggravate these symptoms, but in others they have no effect of that kind.

In regard to the nervous symptoms complained of by those suffering from this disease, their "name is legion;" and you must not accuse them of affecting them at will, if they experience a transition from the most extreme suffering and pain, and cramps, to a condition of calmness, or even of hilarity, in a moment. Both conditions are uncontrollable and unaccountable.

Cephalalgia is, like constipation, nearly a constant symptom of endocervicitis; and the occipital pain, of a burning character, is, perhaps, the most constant; but cephalalgia, confined to a particular place on the head, as the summit, or the temple, should direct your investigations to the uterus. Ladies suffering from endocervicitis will often call your attention to the spinal column, and claim they have spinal disease; but the pain at the lower end of the spine is caused by pressure from within, and that in the dorsal region, and through the sides and stomach, from the derangement of the digestive organs and portal circle. These nervous phenomena are often developed in violent hysterical paroxysms. One of these attacks being the occasion of your first call to see these cases, and if the real condition of the uterus is not recognized and treated, you will have repeated calls, on account of the same symptoms, with no lasting benefit resulting from your treatment. If you would relieve yourself of the opprobrium of the "no account doctor" and feel yourself satisfied, and have your patient satisfied likewise, that she was in the way of ultimate recovery, you must pay attention to, and treat, the uterus. Pain in the anterior crural nerve, and, also, in the sciatic nerve, are quite common, and are dependent upon pressure, instead of reflex action or neuralgia. If this mistake is made, and a corresponding treatment carried out, the result will be unfavorable.

Respiration is very curiously affected in this disease, sometimes. A case I have now under treatment has apparent spasm of the diaphragm, so that the respiration is entirely thoracic; and she thinks she will have to stop breathing entirely.

During these spells she has to be in the strongest current of cold air she can find; and then they last from fifteen minutes to an hour. These symptoms, with various irregular and spasmodic action of the respiratory muscles, together with that peculiar feeling of constriction of the throat, as if a ball was raising up in it, are some of the effects of this disease upon respiration.

A lady spoken of by Prof. W. H. Byford, of Chicago, by heaving up the lower part of her chest, made her friends think she was suffering from violent palpitation; but when asked to hold her breath immediately became contemptuously calm.

At another time, she imitated throbbing of the temples, by sudden contractions of the temporal muscles; and when asked to open her mouth as wide as she could, again subsided. The circulation is often unequal, the extremities being cooler and the head hotter than in health.

In examining one of these patients, some months ago, the tumultuous action of the heart was such that I was afraid of organic disease of that organ; and, upon auscultation, at the same time, having my fingers upon the pulse, at the wrist, some beats of the heart could not be detected, at the wrist. This indicated insufficient valvular force; and for a week I reserved my prognosis, from fear of a fatal result, from lesion of the heart; but when visiting her one day, there were such marked hysterical phenomena that my fears all vanished, and the patient is still alive.

These are some of the prominent symptoms of disease of the uterus, and I have dwelt so long on these general symptoms and sympathetic uterine diseases, that Dr. Hodges' remark, in his work "Diseases of Women," is brought forcibly to mind: "That if this all be true, it is a pity a woman has a womb;" yet, as remarked of old, "the half is not told," is as true in reference to the subject under consideration, as it was of Solomon. Of all these symptoms there is no one that may not occur in a person who has a healthy uterus; but when we find an aggregation of these symptoms, they call your attention to the womb as the cause, and if you do not follow up the investigation to an ocular demonstration, as to whether the womb is healthy or not, you will not have done your whole duty. In so doing, you will be able to so direct your efforts to cure, as to be quite certain of results; and this is of importance, as patients do not like to take medicine without some benefit, and it is not pleasant to me to treat a case a long time, and then find a mistaken diagnosis. This brings us to the consideration of the local symptoms, and in doing so, let me say, the local and general symptoms may bear no relation to one-another, in many cases; for when the general symptoms are violent, the local disease may be slight, and "*vice versa*," according to peculiarity of constitution or temperament.

Leucorrhœa is a strong symptom of uterine, os, and cervical inflammation, and also of ulceration. We do not expect sufficient secretion of mucus, even from a mucous membrane, to inundate the parts and appear externally, and when it does so appear we suspect disease. A male urethra showing a gleety discharge, we say, is an inflamed urethra; and the same may be said of other mucous membranes, the rectum, or the conjunctival mucous membrane. If the leucorrhœal discharge is purulent, or greenish, or yellowish, with fœtor, we conclude ulceration accompanies the inflammation, and is, in a degree, a worse disease. This discharge is known among the ladies as "whites," and appropriately enough too, when it arises simply from inflammation; for as the discharge passes from the womb into the vagina, it is of an albuminous character, and meeting with the acid of the vagina, it is coagulated, and when it appears externally it is of an opaque whiteness, on account of these minute coagula.

When all the acid of the vagina is used up and the discharge continues, it is seen to be a tenacious, glairy mucus; but this should not be mistaken for the vaginal mucus, arising from glandular excitement, that will not discolor the linen, and is a healthy discharge. Quantity of discharge does not correctly indicate extent of disease, for ulcerated surfaces differ in amount, and some secrete none at all, and the rapidity of absorption is different in different cases.

The pains in the illia, loins, groins, and sarcal region are more due to that vague and indescribable condition known as sympathetic than they are to pressure or traction on the uterine ligaments, and easily disappear upon the cure of the inflammation. Hemorrhoids is often caused by the pressure of the uterus, accompanied with constipation upon the hemorrhoidal vessels; and if it has continued a long time will have caused deposition of fibrin or tumors, or other change of structure. The results will not be cured when the uterine inflammation is, but will require special attention afterwards. Ordinarily the sensation of bearing down is on account of the increased weight of the uterus, from inflammation, and loss of tone of the ligaments of the womb, and is no more complained of when the uterine inflammation is cured. Sometimes it is only apparent, and is accounted for by irritability and exalted sensibility, from inflammation; in others there is real displacement, that may require treatment, after the primary disease is cured.

Menstruation is generally affected by uterine inflammation, but not invariably. It is not regular as to time, it being sometimes delayed, and again more frequent than natural. A case under my care last year had not menstruated for five years, but after four months' treatment they (the menses) were reëstablished. Another case had profuse menstruation at the regular time; and, between the periods, bleeding would occur from the slightest disturbance. The function of reproduction is often suspended in ladies suffering from this disease. One of my patients had been married nine years, and had never been pregnant, but after three months' treatment became so, and has now a living child.

Abortions frequently occur in consequence of it, and inflammation is often caused by forced abortions. It is possible, however, for a woman to get pregnant and bear living children, even if she is suffering from inflammation of the womb; but, commonly, it occasions sterility. Endocervicitis is, indeed, a more common cause of sterility than any other.

It is not supposed that this disease affects labor one way or the other, but the effects upon the *post partem* condition are slow involution, a tedious getting up, a longer continuance of the lochial discharge, and more danger of metritis, phlebitis, and pyæmia.

Some physicians have been in the habit of recommending ladies to marry who were suffering frôm this disease, and encouraging them in the belief, that if they became pregnant, they would be cured by it. It may be possible pregnancy has cured this disease, but I do not believe it, and would recommend the cure of the disease previous to pregnancy; for sexual pleasure, indulged in, as it usually is, by newly married people, would not only increase it, but might even produce it.

Some of the causes, besides gross sexual indulgence, are the reading improper books, that keeps up a continual hyperæmia of the sexual organs, till it results in inflammation. Of all the causes, according to my observation, constipation is the worst; and it is strange that so many ladies so utterly neglect this important function, and, by carelessness, contract so bad a habit as to go days, and some of them weeks, and even over a month, without an evacuation of the alimentary canal. I have now under observation a young lady, who has not, on an average, had a movement of the bowels more than once a month, during the last two years, and has gone as long as eight weeks and five days. The same person has been four days and some hours without passing urine, and her menstrual function is irregular. Doubtless she is affected with endocervicitis, but I have never had an opportunity of verifying it by the adequate examination. She is able to be about most of the time, and does not show signs of disorganization sufficient to terminate life soon. Certain kinds of business that make it necessary to be on the feet standing or walking, a long time, are prolific causes of this disease; and, I believe, a pretty constant use of the sewing machine is a cause. Those miserable "Jew devil" appliances, known as abdominal supporters and pessaries are frequent causes. I know of no abdominal supporter but what, by pressure against the abdominal walls, tends to displace the viscera, those below it downwards, and those above it upwards, and, in this way, tend to produce the disease. Use a tight corset and an abdominal supporter, and you have a good combination of causes. If there is any pessary that is worthy of trying at all, it is my opinion Babcock's, and of this I am not prepared to give a verdict, till further proof, but the principle seems to be philosophical. Cold during the menstrual flux, sudden jolts, or severe exercise are causes.

Gonorrhœal vaginitis, by extending to the os and cervix uteri are causes. In regard to prognosis, there appears to be no tendency to a spontaneous cure; and although it may not be necessarily fatal in itself, it is dangerous about the crisis of life, and, doubtless, many women die at that time, who are suffering from this disease, that would not have died if the endocervicitis had been cured. It may be fatal in inducing other disease, and, at all events, the sufferer will never be well, and, yet, at times will be much worse than at others. If one of these patients is provided with the most favorable hygenic influences, her constitutional symptoms will be improved, but her local disease will not be cured, and she will lapse again into bad health. It is possible, some may get well after the turn of life, but some of the most obstinate cases met with have passed that stage.

The prospect of cure, under appropriate treatment, is exceedingly good, but what particular per cent. of cures, I have no means of knowing.

Cure means the healing of all complaints connected with this inflammation, and not alleviation or cure of part. Those cases of hemorrhoidal tumors, and those of hardened or nodulated os, or any case in which there is deposition of fibrin, may be exceptions, or at least require special treatment. These cases, and those in which the inflammation extends through the cervical canal, will be more intractable to treatment than those where the disease is confined to the os. Endocervicitis cannot be cured in less than from three to twelve months; and it is better to tell our patients this than to have them expect to be cured sooner, and be disappointed. Some of them will begin to feel better pretty soon after treatment is commenced, and others not till it is nearly completed, because some remnants of the disease remain, although in process of cure. For instance, when treating chronic ulcer of the leg, it is seen to be healing, but not healed. Young ladies, as a general thing, will respond to treatment sooner than older ones. Persons who have a hereditary predisposition to insanity will be likely to have it excited by this disease, therefore, it would not be safe to promise too much to this class of persons. In order to be certain of diagnosis, it is necessary to see the parts diseased, and to do this, certain instruments are to be used, and the same instruments will be useful in the treatment. They are a speculum, which should be quadrivalve and supplied with a director, that can be withdrawn after the introduction. A pair of dressing-forceps, sufficiently long to easily reach the parts under examination, and a flexible uterine probe or sound, and some cotton. It will be advisable to first examine the parts with the first and second finger, for the purpose of ascertaining the situation, the heat, dryness, tenderness, hardness, and general condition of the parts. Directions for the manner in which this should be done, and also in regard to the introduction of the speculum, can be found in the published works on these diseases, for instance, Prof. W. H. Byford's work, "Medical and Surgical Diseases of Women," and others. After the os is fairly in the mouth of the speculum, the appearance of it as to color, size, and form is to be observed; always bearing in mind that when they are healthy, the color is like that of the mouth (the mouth, that is between the jaws, and contains the teeth, tongue, etc.), and when inflamed is redder, and if ulcerated, is still redder than natural. If the examination is not carefully made, and the secretions thoroughly wiped away with the forceps and a little cotton, we may be deceived as to the true condition of the parts; for the secretions are quite tenacious and obscure the real color of the parts. This shows the condition of the os; and if there is secretion oozing from the cervix, inflammation of the cervical canal may be suspected; and the condition should be determined by examination with the probe, as to the size and sensibility. If, in passing the probe through the cervical canal, a sensation of smarting or rawness is produced, there is ulceration; and if it is found to be much enlarged, hardened, or nodulated it has probably been diseased for a long time. You should also observe the condition of the rectum, at this examination, whether it is loaded with fecal matter or inflamed, as also the bladder and urethra, for they are both likely to be diseased, and, if so, should receive our attention.

The os uteri, in a virgin, has a rounded shape, with a circular opening of small size, or a short slit, with no labia, as in the mother; and in old age, the os seems like a hole at the bottom of a *cul-de-sac.* It is expected that the os uteri and vagina will be moistened with secretion, in a normal condition, but if the parts are profusely smeared or inundated with it, there is disease.

Again, if there is pus mixed with the mucus, there is ulceration as well as inflammation; and the more secretion there is the greater the departure from health. After such an examination, and if such symptoms and conditions are seen, we unhesitatingly say, there is inflammation and ulceration, one or both. If the appearance should be such as to leave any chance for doubt, the application of lunar caustic to the suspected part will relieve it, because an inflamed or ulcerated mucous membrane will instantly turn white under the influence of it, whereas a healthy one will not.

The one disease for which this may be mistaken is cancer, in some of its stages; but the characteristics of cancer, now so well-known, must be your guide. In cancer, when in a schirrous condition, the cervix is hard, unequal, and nodulated. Discharge sometimes absent, in others abundant and serous. Pain in cancer sharp and lancinating. Not unfrequently accompanied with hemorrhage, in the ulcerated stage, and adhesions to other parts. Ulcerations, deep and irregular, with hard edges. These are some of the characteristics of cancer.

When we come to estimate the treatment of disease, we meet with the difficulty of properly estimating the amount that should be credited to nature, and that to the remedial agent used. If these cases, when under the most favorable hygienic circumstances, do not get well, without medical treatment, and do get well when remedial means are used, this is satisfactory evidence that our course of treatment has been beneficial. In no kind of cases has there been better evidence of benefit from treatment than in the one under consideration; and I am as thoroughly convinced that constitutional treatment alone is inadequate. Hygienic means, without other aid, will so fail. Do not understand me to say that they (the hygienic and constitutional) are to be neglected, but, by all means, they are to be secured to the greatest possible extent, and are entitled to credit, to the full amount of benefit they confer. A class of physicians have arrayed themselves against the treatment of endocervicitis, by the local application of caustics; and, I ask, why are they not opposed to the local application of medicine, to granular conjunctivitis, with the same propriety? Why do they not treat the latter disease by constitutional means alone? I suppose they have seen the effects of strong stimulants, in, at first modifying, and then curing, it. All right—so have I, in reference to the treatment of inflammation and ulceration of the womb. We will then make use of all means known to have a favorable effect upon disease, in our course with these cases; and prominent among them shall be local applications, directly to the part diseased, through the agency of the speculum.

A judiciously directed system of exercise, such as riding, walking, or any suitable out-of-door exercise. A well-regulated, nutritious diet; and this should be so directed as to influence the constipation, that so often accompanies this disease. Sexual intercourse must be strictly forbidden, for the irritation consequent upon the act will do more harm than your treatment will do good, subjecting the attendant to failure. There is a particular nervous prostration which occurs in some of these cases, although hæmatosis and nutrition are fairly performed, which will need attention. They will assure you they are so weak they cannot rise from the bed, and that they are ready to commit suicide, and that they know they cannot be cured. It is important that the confidence of such patients is secured, and they be assured that others have been troubled with the same feelings, and are now well, and that she will see all these troubles subside, as she progresses towards cure.

One thing I must say here, in reference to those exceedingly depressing paroxysms of nervousness, often met with in these cases, and that is the effect of cold air. It is truly surprising how soon they will be comfortable if you open all the windows and doors, having the body comfortably covered, and let a draft of cold air pass through the room, clearing it of visitors. The continuance of the cold must be left to the judgment, in each case; but we should advise them to sleep in a cold room and be much out of doors.

In regard to constipation, something has been said, but much more should be said than I have space for. For the most part, persons become constipated by carelessness and inattention, thus forming an irregular habit; the bowels become distended and weakened, and relaxed, thereby perpetuating the condition. In our directions to such persons, we should impress upon them the necessity of forming a regular habit of evacuating the bowels, at a regular time, and that they should let nothing hinder them from attending to it, and that it is as easy to form a regular habit as an irregular one. All that can be done in this way, or by diet, or exercise, or drink, or by the use of our seedy berries and acid fruits, will be better done than by the use of medicines. The use of purgatives, and, particularly, of the thousand and one pills found in every drug-store, are a prolific cause of constipation, and their use is not only unphilosophical but reprehensible. Yet it will often be found necessary to empty the bowels; and if the fæces are uniformly dry, a saline laxative will be indicated, and of these, the sulphate of magnesia is the best. If it is administered with a little sulphuric acid it will not disappoint you.

It may happen here, in the West, that the portal circle is slow, in which case, two grains of calomel or ten grains of blue-mass, taken at bedtime, and followed in the morning with a saline laxative, will be the best, and may be repeated every five days, till the torpidity is removed. For that kind of constipation, dependent upon a loss of tone in the structure of the bowels, the nux vomica is the remedy "*par excellence*," that will do most good; and as anæmia is a frequent concomitant, in these cases, the nux vomica may be usefully combined with iron. Rhei, as an alimentary tonic, is a valuable laxative, and is not open to the objection that it loses its susceptibility upon the bowels, as so many medicines do. A good combination is the following:—

℞. Strychnia,-------------------------------- gr. j.

Ext. Rhei,-------------------------------- ℈ iss.

Sul. Ferri, -------------------------------- gr. x.

Mix. Make into 16 pills, and give one after each meal, or less frequently, as may be thought best; not forgetting to observe the cumulative effects of the strychnia. Or five grains of pul. nux vomica, and one or two grains of sul. quinia, in the same way; and, at the same time, taking the pyrophosphate of iron, in two grain-doses, three times a day. In plethoric cases, the use of bromide of potassa or ammonia will be found useful in controlling nervous excitability.

We will now consider the local means found useful; and prominent among them are the nitrate of silver, caustic potassa, acid nitrate of mercury, tannin, tinct. iodine, liq. ferri per sulphas, and some other stimulating astringents. There is no doubt, the nitrate of silver will suit, generally, the best of any of them, and is the one usually resorted to first. For a port-caustique, the holder should be flexible, so as to be made to conform to the different curvatures of the cervix; for it will be necessary to reach the whole cervical canal, in case the disease is thus extensive. The piece of caustic should be half an inch in length, of the cylindrical sticks found in the shops; and after introducing the speculum and getting the os uteri nicely in it, the secretions are to be carefully wiped away with the dressing-forceps and a little cotton, and all the inflamed and ulcerated surfaces deliberately touched with caustic. This is to be repeated in from five to seven days, while a vestige of disease is left, except during the menstrual flux. Some cases will bleed upon the slightest disturbance, in which case, the liq. ferri per sulphas may be used on a swab of cotton, to commence with, once in four days, till the tissues are condensed, and then the caustic, to complete the cure. The nitrate may fail, in some cases, or do harm, and have to be abandoned. Why? is not easy always to explain. It may not be strong enough to arouse the capillaries to action, and we be compelled to resort to something more powerful. The caustic potassa is one, and the acid nitrate of mercury is another, that may be tried; and after one or two applications, a month apart, the nitrate of silver may be again used with benefit.

During the whole treatment, a system of bathing should be carried out, for the purpose of cleanliness, and to reduce the local congestions. The sitz bath, of a temperature agreeable to the patient, can be used, at least twice a day, and three or four times, in some cases, with benefit. A womb bath is also useful. It may be used by having two vessels, one with six or eight quarts of water in it, of a suitable temperature; the water may be thrown into the vagina with an extension syringe, and allowed to run into the empty vessel. These baths wash away the secretions and reduce the inflammation. For the purposes of cleanliness, a general bath should be used, two or three times a week. The water should be tepid at first, and used gradually cooler and cooler, till it is used cold. Astringent injections into the vagina are useful, through the whole treatment. A suitable one is, water, one quart, and alum, one drachm. The length of time which should intervene between these injections will vary somewhat, but should never be repeated till the parts have become uniformly moist, for some hours.

A weak solution of sul. zinc, or copper, or nitrate of silver may be used for a vaginal injection. It will always be advisable to use water to wash away the secretions, before using the medicated injection, so that the astringent may come in contact with the diseased mucous surfaces, in its full strength. It may happen that in using the vaginal injection, with a tube having several perforations, that one of them is opposite the mouth of the womb, allowing the water to enter the neck and pass along into the uterus. This produces spasmodic pain in the womb, perhaps, for the purpose of expelling it; and these pains extend to the back, the person's extremities become cold, with sickness of stomach, making quite an alarming array of symptoms.

Such symptoms will readily subside if an opiate is given and hot fomentations applied to the abdomen. During treatment with the nitrate of silver, the os and cervix contracts, so as to prevent the free discharge of the menstrual or other secretions, thereby causing pain. Bougies may be used to dilate the cervical canal, and they also exert a favorable influence upon the inflammation, by pressure upon the capillaries.

These bougies may be made of gum, or slippery elm, or metal, or sponge. A piece of slippery elm bark, cut two inches long, and of the proper size, having a thread tied to it long enough to reach out externally, may be introduced into the cervix, and allowed to remain 24 hours. This should be repeated every five days, as long as necessary. Medicines can be introduced in this way, if thought best; and the first appropriate case I see, I mean to use, in this way, carbolic acid in glycerine, confidently expecting favorable results from it.

In very aged persons, the nitrate will make them worse, as the inflammation assumes a peculiar character, the granulations being extremely minute, and of a bright scarlet color. The potassa fusa or acid nitrate of mercury applied a few times, at intervals of a month, and then followed by applications of solid sulphate of copper, will be better.

Submucous inflammation, in connection with mucous inflammation and ulceration, is a frequent complication, and deserves some notice. If there is unusual tenderness, upon pressure with the probe or finger, of the cervix, and if heat and swelling accompanies the tenderness, the submucous inflammation will require separate attention. If there is not much tenderness or swelling, it will subside when the mucous inflammation is cured, by the means formerly indicated. The special means used for the submucous inflammation are, leeches, alteratives of calomel, followed by sul. magnesia or Seidlitz powders; and if leeches are not at hand, scarifications may be substituted for them.

These means, together with the thorough use of the sitz and womb baths, will generally meet your expectations. When I commenced this paper, I designed giving some cases in detail, but it has been drawn out to such unusual length, I forbear. If the course here indicated is thoroughly carried out, the profession will all have detailed cases of cures to relate; for, I am sure the disease, although not disposed to spontaneous cure, can be cured in this way. So confident am I, that when I hear of an apparent failure, I attribute it to lack of thoroughness. In conclusion, if the members of the Society and the profession are induced to give this class of sufferers a more careful and considerate attention, my object will be gained, and I feel repaid for my trouble.

BUDA, *December 4th, 1869.*

